# Cost-effectiveness of cardiovascular magnetic resonance and single-photon emission computed tomography for diagnosis of coronary artery disease in Germany

**DOI:** 10.1186/1532-429X-15-30

**Published:** 2013-04-10

**Authors:** Julia Boldt, Alexander W Leber, Klaus Bonaventura, Christian Sohns, Martin Stula, Alexander Huppertz, Wilhelm Haverkamp, Marc Dorenkamp

**Affiliations:** 1 Department of Cardiology, Charité – Universitätsmedizin Berlin, Campus Virchow-Klinikum Augustenburger Platz 1, Berlin 13353, Germany; 2 Department of Cardiology, Schulich Heart Centre, Sunnybrook Health Sciences Centre, University of Toronto, Toronto, Canada; 3 Department of Cardiology, Angiology, and Conservative Intensive Care, Klinikum Ernst von Bergmann, Potsdam, Germany; 4 University Outpatient Clinic Potsdam, Sports Medicine and Sports Orthopaedics, University of Potsdam, Potsdam, Germany; 5 Department of Cardiology and Pneumology, Heart Center, Georg-August-Universität Göttingen, Göttingen, Germany; 6 HELIOS Medical Care Center Weimar, Cardiologist and Center Director, Weimar, Germany; 7 Imaging Science Institute Charité, Berlin, Germany; 8 Department of Radiology, Charité – Universitätsmedizin Berlin, Berlin, Germany

**Keywords:** Cost-effectiveness, Cardiovascular magnetic resonance, Scintigraphy, Coronary angiography, Coronary artery disease

## Abstract

**Background:**

Recent studies have demonstrated a superior diagnostic accuracy of cardiovascular magnetic resonance (CMR) for the detection of coronary artery disease (CAD). We aimed to determine the comparative cost-effectiveness of CMR versus single-photon emission computed tomography (SPECT).

**Methods:**

Based on Bayes’ theorem, a mathematical model was developed to compare the cost-effectiveness and utility of CMR with SPECT in patients with suspected CAD. Invasive coronary angiography served as the standard of reference. Effectiveness was defined as the accurate detection of CAD, and utility as the number of quality-adjusted life-years (QALYs) gained. Model input parameters were derived from the literature, and the cost analysis was conducted from a German health care payer’s perspective. Extensive sensitivity analyses were performed.

**Results:**

Reimbursement fees represented only a minor fraction of the total costs incurred by a diagnostic strategy. Increases in the prevalence of CAD were generally associated with improved cost-effectiveness and decreased costs per utility unit (ΔQALY). By comparison, CMR was consistently more cost-effective than SPECT, and showed lower costs per QALY gained. Given a CAD prevalence of 0.50, CMR was associated with total costs of €6,120 for one patient correctly diagnosed as having CAD and with €2,246 per ΔQALY gained versus €7,065 and €2,931 for SPECT, respectively. Above a threshold value of CAD prevalence of 0.60, proceeding directly to invasive angiography was the most cost-effective approach.

**Conclusions:**

In patients with low to intermediate CAD probabilities, CMR is more cost-effective than SPECT. Moreover, lower costs per utility unit indicate a superior clinical utility of CMR.

## Background

Coronary artery disease (CAD) is a major cause of death and disability in developed countries and can be considered as a global public health challenge [1,2]. Given its prevalence and related morbidity, the total economic burden of CAD is enormous [3]. Diagnostic strategies that allow an early and accurate diagnosis of CAD are therefore highly desirable, both medically and economically [4].

Imaging tests have significantly improved the detection of CAD, with single-photon emission computed tomography (SPECT) being one of the most commonly used methods [5]. Although widely available, SPECT has its limitations, such as a relatively low spatial resolution and patient exposure to ionizing radiation [6]. In recent years, cardiovascular magnetic resonance (CMR) has emerged as an important imaging modality for the non-invasive assessment of CAD [7-9]. Potential advantages of CMR include the lack of ionizing radiation, high spatial resolution, and versatile imaging capabilities which allow visualizing different pathological aspects of CAD during a single patient examination (e.g. myocardial viability, cardiac function and morphology) [10,11]. Earlier studies have evaluated the diagnostic accuracy of stress-perfusion CMR for the detection of CAD and some studies have reported equal or improved results compared with SPECT [12-16]. However, the findings of a recent trial, which exploited the full potential of CMR, demonstrated a superior diagnostic accuracy of CMR compared with SPECT [17].

As the costs of CMR may significantly exceed the costs associated with SPECT, the economic effect of CMR must be considered [18]. The present study therefore aimed to determine the cost-effectiveness of CMR as an alternative to SPECT in patients with suspected CAD.

## Methods

### Study design

Based on Bayes’ theorem, a previously described mathematical model was used to determine the comparative cost-effectiveness and utility of the following three approaches for diagnosing CAD: (1) CMR, (2) SPECT, and (3) invasive coronary angiography [19-21]. The effectiveness data and other model input parameters were derived from the literature.

### Effectiveness of tests

Effectiveness of diagnostic tests was defined in two ways. The first effectiveness criterion was the ability of a diagnostic test to accurately identify a patient with CAD [19-22]. This definition represents a straightforward approach assuming that the single goal of a test is to make a diagnosis [19-21]. The definition of the second effectiveness criterion was more complex and attempted to account for the future health outcome of patients undergoing the tests (i.e., clinical utility) [19,20]. It was assumed that a correct diagnosis of CAD would enable patients to receive optimal therapy resulting in improved survival and well-being. Over the follow-up period, the number of life-years gained (Δ) by CAD therapy was adjusted for quality of life, yielding quality-adjusted life-years (QALYs). By convention, utility or quality of life scores are expressed on a scale from 0 to 1, with 0 being equivalent to death and 1 being a state of perfect health. QALYs are calculated by multiplying the length of time spent in a particular disease state by the utility associated with that disease state. In line with previous cost-effectiveness analyses, an accurate diagnosis of CAD was projected to increase the number of QALYs by 3 years during a 10-year follow-up [20]. Equations and details of the underlying calculations can be found elsewhere [19,20]. The follow-up was limited to 10 years for purposes of conservative estimation [19,20]. Importantly, the computed increase in QALYs (ΔQALY) is only used as a common denominator to facilitate a comparison of cost per utility unit of different tests to diagnose CAD [19,20]. Rather than calculating the absolute cost of each diagnostic test, the present study was designed to compare the rank order of cost per utility unit of the evaluated tests [19,20].

### Diagnostic procedures

It was assumed that CMR was performed on a 1.5 Tesla scanner and that the imaging protocol consisted of myocardial rest and adenosine stress perfusion imaging, late gadolinium enhancement, and left ventricular cine imaging [17]. Data on the diagnostic accuracy of CMR were derived from the recently published CE-MARC (Clinical Evaluation of Magnetic Resonance imaging in Coronary heart disease) trial, after excluding non-invasive coronary angiography data from the analysis (Table 1) [17]. The CE-MARC trial included 752 patients with an estimated prevalence of significant CAD of 40-60%. All patients were scheduled for CMR and SPECT, followed by invasive coronary angiography [17]. The rate of severe complications related to CMR was taken from the EuroCMR (European CMR) registry [23]. Due to the lack of data, the procedure-related mortality rate of CMR was assumed to be the same as for exercise stress testing [19,20,24]. In the base case scenario, the rate of non-diagnostic CMR examinations was set at 5% [17,25].

**Table 1:** Model input parameters

**Parameter**		**Value**	**Range**	**Ref.**
Sn_C_	Sensitivity of CMR	0.82	0.77-0.86	[17]
Sp_C_	Specificity of CMR	0.86	0.82-0.89	[17]
Sn_S_	Sensitivity of SPECT	0.67	0.60-0.72	[17]
Sp_S_	Specificity of SPECT	0.83	0.79-0.86	[17]
Sn_A_	Sensitivity of angiography	1.00	N/A	[19-21]
Sp_A_	Specificity of angiography	1.00	N/A	[19-21]
R_C_	Complication rate with CMR	0.0005	0.0001-0.001	[23]
R_S_	Complication rate with SPECT	0.0005	0.0001-0.001	[20]
R_A_	Complication rate with angiography	0.005	0.001-0.01	[28]
R_F_	Complication rate for patients with CAD and false-negative test results*	0.25	0.15-0.30	[20,21]
M_C_	Mortality rate due to CMR	0.00005	0.00001-0.0001	[20,24]
M_S_	Mortality rate due to SPECT	0.00005	0.00001-0.0001	[20,24]
M_A_	Mortality rate due to angiography	0.00075	0.0001-0.0015	[28]
M_F_	Mortality rate for patients with CAD and false-negative test results*	0.20	0.15-0.25	[19,20]
NDx_C_	Rate of non-diagnostic CMR	0.05	0.01-0.1	[17,25]
NDx_S_	Rate of non-diagnostic SPECT	0.05	0.01-0.1	[17,25]
NDx_A_	Rate of non-diagnostic angiography	0.00	N/A	[19-21]
C_C_	Cost of CMR [in €]	703	527-879	[31,34]
C_S_	Cost of SPECT [in €]	504	378-630	[30,33]
C_A_	Cost of angiography [in €]	2,926	2,195-3,658	[29,35]
C	Cost of a complication^#^ [in €]	14,478	10,859-18,098	[21,36]
N_C_	No. of patients having CMR	1.0	N/A	[21]
N_S_	No. of patients having SPECT	1.0	N/A	[21]
N_A_	No. of patients having angiography	varies	Equation 1a	[19-21]
NF_C_	No. of false-negative CMR	varies	Equation 1a	[19-21]
NF_S_	No. of false-negative SPECT	varies	Equation 1a	[19-21]
P	Prevalence of CAD	varies	0.1-1.0	[19-21]
ΔQALY’	QALY extended by CAD therapy*	3.0	2.0-4.0	[20]
ΔQALY	Net QALY gained	varies	Equations 1c,2c	[20]

*Over a 10-year follow-up period.

^#^Assumed to be myocardial infarction (or cerebral, for invasive angiography).

Angiography refers to invasive coronary angiography. Details of the parameters are given in the text.

CAD: coronary artery disease; CMR: cardiovascular magnetic resonance; N/A: not applicable; QALY: quality-adjusted life-year; SPECT: single-photon emission computed tomography.

In line with the CE-MARC trial, SPECT imaging was assumed to be performed with a dual-head gamma camera using a standard 99mTechnetium-based 2-day protocol, as advised in current guidelines [17,26,27]. Rest and stress electrocardiographically (ECG)-gated SPECT images were acquired using an adenosine protocol identical to the one used for CMR [17]. As for CMR, the diagnosis of CAD incorporated all available data (i.e., perfusion during rest and stress conditions, cardiac wall motion analysis, and left ventricular volumes). Overall accuracy of SPECT for the diagnosis of CAD was again taken from the CE-MARC trial [17]. Data on serious complications and mortality due to SPECT were derived from previously published cost-effectiveness analyses on cardiac SPECT imaging [19,20,24]. The rate of non-diagnostic examinations was assumed to be the same as for CMR imaging [17,20,25]. Detailed descriptions of both the CMR and SPECT imaging protocols are given elsewhere [17].

Invasive coronary angiography using standard techniques constituted the standard of reference. Clinically significant CAD was defined as the presence of a coronary stenosis of 70% or more in at least one major coronary artery, or 50% stenosis in the left main stem [17]. By definition, invasive angiography represented a perfect diagnostic test (i.e., 100% sensitive and 100% specific) without any non-diagnostic results [19-21]. The rates of complication and mortality for patients undergoing invasive coronary angiography were derived from the literature [28].

### Costs

Economic analyses were conducted from the perspective of a health care payer (German health care insurance system). Cost data were obtained from multiple sources, including the 2012 version of the German Diagnosis Related Groups (G-DRG) system, the Uniform Value Scale (*Einheitlicher Bewertungsmaßst*ab, EBM), and the German doctor’s fee schedule (*Gebührenordnung für Ärzte*, GOÄ) (Table 1) [29-31]. Both, SPECT and CMR were considered as outpatient tests, while invasive coronary angiography was considered an inpatient procedure [18]. According to the EBM catalogue, and a monetary point value of €0.032, the costs of SPECT amounted to €504 (EBM reimbursement codes 17210, 17330, 17331, 17333, 17363, 40520, and 40522) [30,32,33]. In the EBM system, CMR cannot yet be coded as a specific examination and costs of CMR are not covered adequately with existing reimbursement options for thoracic magnetic resonance imaging (€204 including a lump sum of €50 for contrast agent) [18,34]. Thus, we opted to calculate the costs of a CMR study according to the GOÄ system (GOÄ reimbursement codes 200, 271, 346, 347, 602, 5715, 5731, and 5733) [31,34]. The basic costs given in the GOÄ catalogue can be multiplied by a specific factor, for instance up to 1.8 for radiological examinations, if the procedure was more complicated than average or if both the patient and physician have agreed beforehand that a multiplication factor will be applicable [31]. For calculation purposes, averages of the basic and the maximum costs were taken for each GOÄ code. Including a fee of €150 for material costs, CMR costs totaled €703 (Table 1). Costs for invasive coronary angiography were calculated as the average of the G-DRGs F49B, F49E, and F49G with a base rate of €2,970 (Table 1) [29,35]. Conservative cost estimates for a major complication amounted to €14,478 [21,36]. All costs were given in Euro (€) and were rounded to the nearest whole amount.

### Calculation of cost-effectiveness and utility

Total costs were calculated by multiplying direct costs with the number of patients tested plus the induced costs. Cost-effectiveness was calculated as cost per effect [19-21]:

$$\frac{\mathrm{Direct}\;\mathrm{Costs}+\mathrm{Induced}\;\mathrm{Costs}}{\mathrm{Effectiveness}}$$

**Figure 1:**
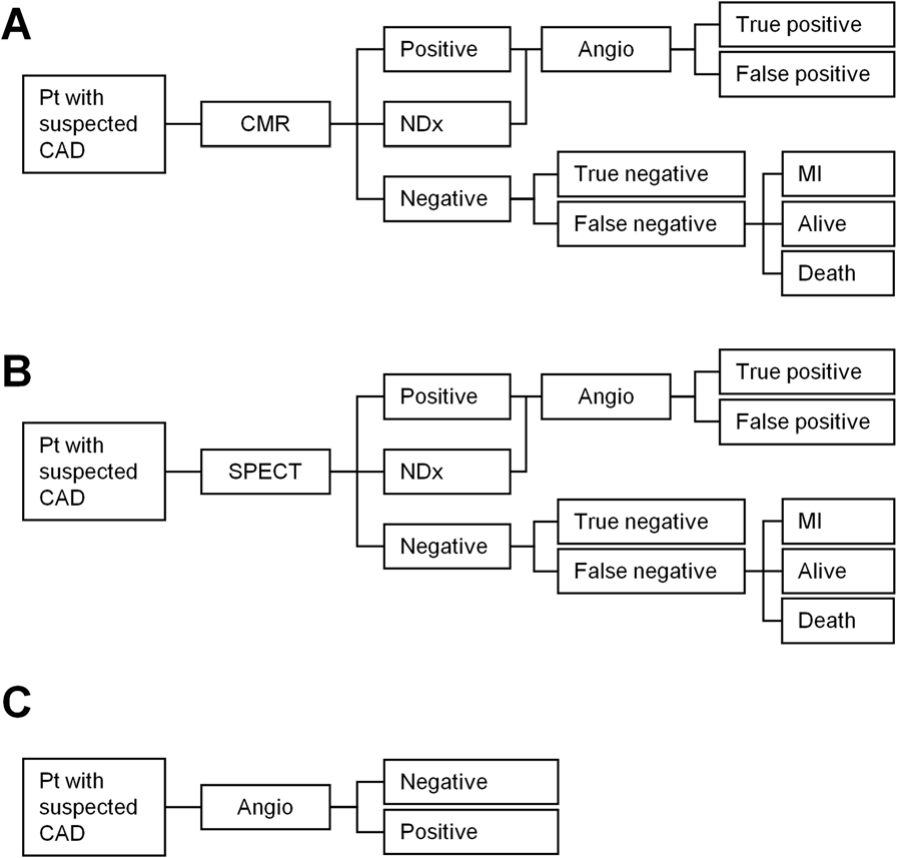
**Diagnostic algorithms.** Diagnostic algorithms for patients (Pt) presenting with suspected coronary artery disease (CAD). In algorithm **A**, cardiovascular magnetic resonance (CMR) was performed first and patients were referred for invasive coronary angiography (Angio) only if CMR was positive or non-diagnostic (NDx). Patients with false-negative test results were at risk for myocardial (MI) or death from undetected CAD. Diagnostic algorithm **B** had the same basic structure as algorithm **A**, but involved single-photon emission computed tomography (SPECT) instead of CMR. Algorithm **C** used invasive coronary angiography as the first and only test to diagnose CAD.

In this respect, direct costs reflected the fees for diagnostic tests, as given in Table 1. Induced costs included cost of complications associated with the test procedures or false-negative CAD results multiplied by the number of patients tested as well as subsequent testing triggered by the results of the first test (e.g. invasive coronary angiography after a positive result of CMR or SPECT) [19-21]. Effectiveness was a patient with CAD diagnosed. The utility of a test was its ability to add a number of QALYs over a 10-year follow-up period [19,20]. In the diagnostic algorithms involving CMR (Figure 1A) or SPECT (Figure 1B), CMR or SPECT were performed first and were followed by invasive angiography but only if non-invasive testing showed positive results or was non-diagnostic [21]. Specifically, calculations of cost-effectiveness and utility involved the equations below (Table 1 shows parameters used in equations) [19-21]:

1. CMR (or SPECT)

a) *Costs* = *N*_*C*_ · (*C*_*C*_ + *R*_*C*_ · *C*) + *N*_*A*_ · (*C*_*A*_ + *R*_*A*_ · *C*) + *NF*_*C*_ · (*R*_*F*_ · *C*)

whereas

*N*_*A*_ = *N*_*C*_ · (1 − *NDCx*_*C*_) · [*P* · *Sn*_*C*_ + (1 − *P*) · (1 − *Sp*_*C*_)] + *N*_*C*_ · *NDx*_*C*_ and *NF*_*C*_ = *N*_*C*_ · (1 − *NDx*_*C*_) · *P* · (1 − *Sn*_*C*_)

a) *Effectiveness* = *N*_*C*_ · (1 − *NDx*_*C*_) · *P* · *Sn*_*C*_ + *N*_*C*_ · *P* · *NDx*_*C*_

a) *ΔQALY* = (*CAD*_*Dx*_) · (*ΔQALY* ') − 10 · (*N*_*C*_ · *M*_*C*_ + *N*_*A*_ · *M*_*A*_) − 5 · (*NF*_*C*_ · *M*_*F*_) − 10 · (0.1) · (*N*_*C*_ · *R*_*C*_ + *N*_*A*_ · *R*_*A*_ + *NF*_*C*_ · *R*_*F*_)

Equations 1a-c were used for both CMR and SPECT. For space reasons, only the CMR equations are shown. Application to SPECT required replacing CMR-specific with SPECT-specific variables (Table 1). Equation 1c, the formula to calculate net QALYs gained (ΔQALY), assumed that deaths caused by diagnostic tests subtract 10 years, and deaths due to false-negative test results and hence missed CAD subtract 5 years on average [20]. Complications caused by diagnostic testing or by missed CAD were assumed to reduce the quality of life by 1/10 per annum.

In the final diagnostic algorithm, invasive coronary angiography was the first and only diagnostic test to evaluate patients for suspected CAD (Figure 1C). The following equations were applied (parameters are given in Table 1) [19-21]:

2. Invasive coronary angiography

a) *Costs* = *N*_*A*_ · (*C*_*A*_ + *R*_*A*_ · *C*) whereas *N*_*A*_ = 1.0

a) *Effectiveness* = *N*_*A*_ · *P*

a) *ΔQALY* = *N*_*A*_ · *ΔQALY* ' · *P* − 10 · *N*_*A*_ · *M*_*A*_ − *N*_*A*_ · *R*_*A*_

### Data and sensitivity analysis

A one-way sensitivity analysis was performed to evaluate whether the results of the base case scenario were affected by changes in the model input parameters. The variables were changed over the ranges given in Table 1. Most important, the diagnostic sensitivities and specificities of CMR and SPECT were increased and decreased according to the 95% confidence intervals given in the CE-MARC trial [17]. All costs were varied by 25% in each direction (Table 1) [37]. Rates of non-diagnostic CMR and SPECT examinations were changed to 1% and 10%, respectively. The impact of different clinical outcomes on model results was analyzed by varying complication rates, 10-year follow-up estimates, and the value of ΔQALY (Table 1). All analyses were performed with Excel for Windows (Microsoft Office 2010, Microsoft Corp., Redmond, WA, USA).

## Results

### Impact of CAD prevalence on total costs, cost-effectiveness and utility

**Figure 2:**
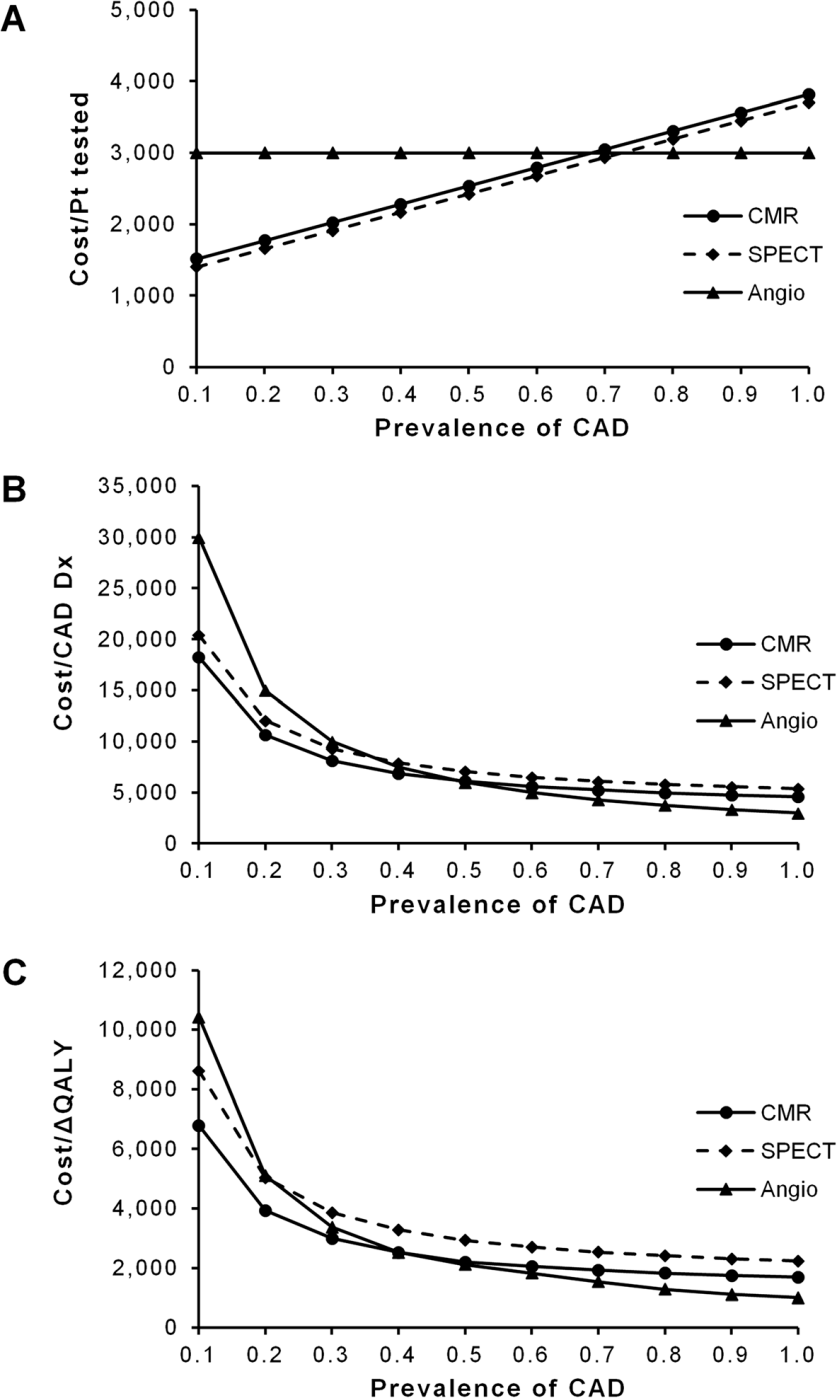
**Effect of CAD prevalence on costs, cost-effectiveness and utility.** In each of the three graphs, prevalence of coronary artery disease (CAD) increases along the horizontal axis. In all cases, costs are given in Euro. The upper graph (**A**) shows the total cost per patient (Pt) tested on the vertical axis for cardiovascular magnetic resonance (CMR), single-photon emission computed tomography (SPECT), and invasive coronary angiography (Angio). Costs increase significantly with CAD prevalence for CMR and SPECT, but not for invasive angiography. Cost per effect, in terms of cost per accurate diagnosis (Dx) of CAD, is depicted in the middle graph (**B**). The decrease of cost per effect with growing CAD prevalence indicates growing cost-effectiveness as cost per effect is the inverse of cost-effectiveness. The lower graph (**C**) plots cost per utility unit, in terms of quality-adjusted life-years gained (ΔQALY), on the vertical axis. Cost per utility unit decreases as prevalence of CAD increases.

Direct costs of CMR and SPECT, which were reflected by the reimbursement fees given in Table 1, represented only a minor fraction of the total costs incurred by the respective diagnostic strategy (Figure 2A). Prevalence of CAD was varied between 0.10 and 1.0 in increments of 0.10. When set in relation to different prevalences of CAD, total costs of CMR and SPECT increased as a linear function of disease prevalence (Figure 2A). In contrast, costs did not increase significantly for invasive coronary angiography. At a CAD prevalence <0.70, both non-invasive tests were associated with lower total costs than invasive coronary angiography but with higher costs at higher prevalences of CAD (≥0.70). Figure 2B plots cost per effect (defined as accurate diagnosis of CAD) versus prevalence of CAD. All three diagnostic strategies exhibited hyperbolic decreases in cost per effect with increasing CAD prevalences. Because cost per effect is the inverse of cost-effectiveness, this decrease indicates increased cost-effectiveness. Likewise, as the prevalence of CAD increased, there were decreased costs per utility unit in terms of QALYs gained (Figure 2C) Δindicating increased cost-utility at higher disease prevalences. Thus, despite the fact that total costs increased with increasing prevalence of CAD (Figure 2A), cost per effect and cost per utility improved. The hyperbolic relationship between CAD prevalence and cost per effect or cost per utility implicates very high costs per effect or utility unit at low disease prevalences (Figure 2B and C).

### Comparison of diagnostic strategies – CMR versus SPECT

Differences between CMR and SPECT with respect to cost per effect and cost per utility unit were analyzed at varied prevalences of CAD. Results of these comparisons are exemplified for low (0.20), intermediate (0.50), and high (0.80) disease prevalences in Table 2. In populations with a low prevalence of CAD (e.g. 0.20), CMR is the most cost-effective diagnostic approach (i.e. lowest cost per effect), followed by SPECT and with a significant difference by invasive coronary angiography (Figure 2B). At a disease prevalence of 0.50, CMR remained the most cost-effective approach, while SPECT became least cost-effective and the differences among the three diagnostic strategies were less dramatic. In a population with a high CAD prevalence (e.g. 0.80), invasive coronary angiography was most cost-effective. However, the rank order between CMR and SPECT never changed (Figure 2B) and CMR was always more cost-effective and exhibited lower costs per effect than SPECT (indicated by minus-signs in Table 2). At a low prevalence of CAD (0.20), the rank order of cost per utility unit was principally the same as that of cost per effect (Figure 2C). Again, the rank order of tests changed at an intermediate disease prevalence and performing invasive coronary angiography as the first and only test was the most cost-effective diagnostic approach at high disease prevalences (e.g. 0.80) (Figure 2C). Despite lower direct costs (i.e., fee for diagnostic testing; Table 1), SPECT was always linked to higher total costs per utility unit than CMR (denoted by minus-signs in the far right column in Table 2). The similarity of the graphs displaying cost per effect (Figure 2B) and cost per utility unit (Figure 2C) is indicative of the robustness of the employed mathematical model.

**Table 2:** Health and economic outcomes

					**Difference between CMR and SPECT**	
**Prevalence of CAD**	**Test**	**Total cost (€)**	**Effect (CAD Dx)**	**Utility (ΔQALY)**	**Cost per effect (€/CAD Dx)**	**Cost per utility (€/ΔQALY)**
0.20	CMR	1,770	0.17	0.45		
	SPECT	1,657	0.14	0.33	−1,394	−1,106
0.50	CMR	2,537	0.41	1.13		
	SPECT	2,425	0.34	0.83	−945	−685
0.80	CMR	3,304	0.66	1.81		
	SPECT	3,193	0.55	1.33	−811	−563

CAD: coronary artery disease; Dx: accurate diagnosis (of CAD); CMR: cardiac magnetic resonance; QALY: quality-adjusted life-year; SPECT: single-photon emission computed tomography.

### Sensitivity analyses

**Figure 3:**
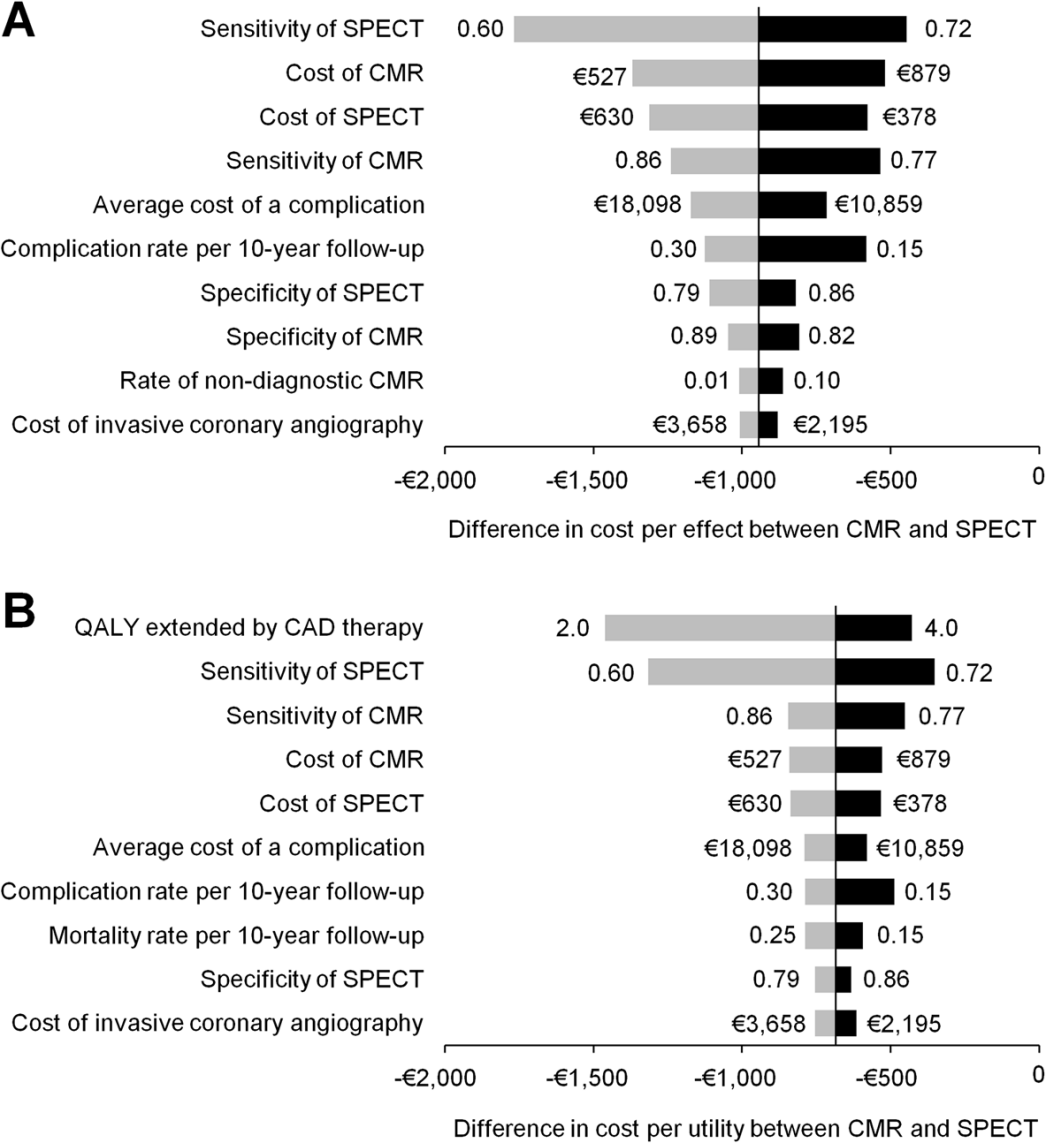
**Sensitivity analyses.** Tornado diagrams displaying the results of deterministic one-way sensitivity analyses of cost per effect (**A**) and cost per utility unit (**B**) determined at a prevalence of coronary artery disease (CAD) of 0.50. Each bar represents a sensitivity variable. The vertical axis intersects the horizontal axis at the base-case cost per effect (−€945/accurate diagnosis of CAD) or cost per utility unit (−€685/increase in quality-adjusted life-years; ΔQALY) difference between cardiovascular magnetic resonance (CMR) and single-photon emission computed tomography (SPECT). The width of each horizontal bar illustrates the impact of the respective parameter on base-case results. The values adjacent to either side of a bar represent the highest or lowest value simulated for each model input parameter. Complication or mortality rates per 10-year follow-up apply to patients with false-negative test results. For input variables, see Table 1.

Assuming base-case values and given a CAD prevalence of 0.50, CMR was associated with total costs of €6,120 for one patient correctly diagnosed as having CAD and with €2,246 per ΔQALY gained versus €7,065 and €2,931 for SPECT, respectively. Differences were calculated by subtracting the results for SPECT from those for CMR and amounted to -€945 per effect (accurate diagnosis of CAD) and to -€685 per utility unit (ΔQALY) (Table 2). These base-case results were robust to plausible alternative scenarios as sensitivity analyses showed that CMR would remain more cost-effective than SPECT through the whole range of parameter estimates (Table 1). Figure 3 presents the results of deterministic one-way sensitivity analyses performed at a prevalence of CAD of 0.50. For clarity, the results are graphically displayed through the use of tornado diagrams. Shown are the 10 parameters that influence the base-case results most, arranged from top to bottom according to their importance. Figure 3A illustrates that the sensitivity of SPECT, costs of SPECT and CMR, the sensitivity of CMR, and the average cost of a complication were the most influential parameters on the cost per effect difference between CMR and SPECT. The results of the sensitivity analysis to investigate the impact of parameter variation on the cost per utility difference between CMR and SPECT are shown in Figure 3B. Base-case results were most sensitive to the amount of QALYs extended by CAD therapy over a 10-year follow-up period, the sensitivities of SPECT and CMR, costs of SPECT and CMR, and the average cost of a complication. For both cost per effect and cost per utility unit, results were least sensitive to variations in the values of complication rates with SPECT and CMR (data not shown). Nonetheless, for all parameters tested in the sensitivity analysis, the differences in cost per effect and cost per utility unit can be considered as economically attractive in favor of CMR. Sensitivity analyses also indicated that above a threshold value of CAD prevalence of 0.60, proceeding directly to invasive angiography was the most cost-effective approach.

## Discussion

### Main findings

To the best of our knowledge, this is the first study to systematically evaluate the cost-effectiveness of CMR compared with SPECT for diagnosing CAD in patients suspected of having this disease. Calculations were performed using a well-validated model based on the equations of Bayes’ theorem and the cost-analysis was conducted from a health care payer’s perspective. Compared to SPECT, CMR resulted in relatively better cost-effectiveness and utility across all prevalence levels and the full range of sensitivity analyses. Cost-effectiveness was influenced mostly by varying the sensitivities of CMR and SPECT, the costs associated with both diagnostic tests, and the average cost of a complication. The same factors, supplemented by the amount of QALYs extended by CAD therapy over a 10-year follow-up period, had the largest impact on cost per utility differences between CMR and SPECT. Above a threshold value of CAD prevalence of 0.60, proceeding directly to invasive angiography was found to be the most cost-effective diagnostic strategy.

### Assessment of cost-effectiveness and utility

In the past years, cost considerations have become increasingly relevant in clinical decision making [38]. Cost-effectiveness analysis can help decision-makers to allocate limited resources and can guide the utilization of latest-generation and presumably high-cost imaging modalities [39].

One of the key requirements of cost-effectiveness analysis is the identification of all relevant costs. If the analysis is carried out from the viewpoint of a health care payer, procedure costs usually represent charges or reimbursement fees [40]. The German health care system is characterized by the presence of public and private health insurers. Within the public system, outpatient health care services such as SPECT are to be charged according to the EBM system [30]. In the absence of a specific EBM procedure code for CMR, we chose to use the GOÄ fee schedule for privately insured patients. This is common practice in Germany, as the GOÄ fees much better reflect the actual costs incurred by CMR [31,34]. As a result, CMR was associated with nearly 40% higher costs than SPECT (Table 1). Importantly, this cost ratio is very similar to cost ratios described for the United States or the United Kingdom. Because our model depends on relative rather than on absolute costs, the results of the present study can be assumed to be generally valid for other health care systems as well [18]. As the study takes a health care payer’s and not a societal perspective, other costs such as lost productivity due to missed days at work (indirect costs) were not included [21,40]. Rather than assessing the impact of diagnostic tests on the overall welfare of society, the goal of the study was to compare the cost-effectiveness and utility of CMR and SPECT to achieve the same objective, i.e. diagnosing CAD and thereby improving the clinical outcome [20,21]. The outcome variable was limited to a 10-year follow-up underlining the conservative nature of our analysis. If the effects of CAD therapy would have been simulated beyond the 10-year horizon, than the impact on outcome (ΔQALY) might have been even more favourable than indicated by our results.

The diagnostic accuracy of CMR to detect CAD was taken from the recently published CE-MARC study which prospectively evaluated the role of CMR in patients with suspected CAD [17]. By comparison, CMR delivered a higher sensitivity and specificity than SPECT [17]. Another large study, MR-IMPACT II (Magnetic Resonance Imaging for Myocardial Perfusion Assessment in Coronary artery disease Trial II), also detailed the accuracy of CMR compared with SPECT [25,41]. MR-IMPACT II confirmed CMR’s position as alternative to SPECT with higher sensitivity to detect CAD. Unlike CE-MARC, the specificity of CMR was inferior to SPECT in MR-IMPACT II [25,41]. However, CE-MARC had a more rigorous study design and included a larger patient population [17,42]. Moreover, CE-MARC used the full potential of CMR, including perfusion imaging, late gadolinium enhancement, left ventricular cine imaging, and non-invasive coronary angiography while MR-IMPACT II focused solely on perfusion abnormalities [17,25,43]. Because coronary angiography by CMR cannot be regarded as standard part of routine examinations and because it is not feasible in all patients, it was not incorporated in our analysis (Table 1). This exclusion led to a slightly diminished diagnostic accuracy of CMR, again reflecting the conservative estimates used in our study [17]. It is noteworthy, that future imaging protocols may even further increase the diagnostic accuracy of CMR [44]. Dobutamine stress CMR, while clinically valuable, was not part of our analysis [45]. For consistency and comparability, data on diagnostic accuracy of SPECT were also derived from the CE-MARC study [17]. Although other studies have reported different and wide varying diagnostic accuracies of SPECT, the superior sensitivity of CMR in comparison to SPECT seems to be a common finding [17,25,41,46]. With respect to cost-effectiveness and utility, our results clearly indicate that a high sensitivity is more important than a high specificity (Figure 3A and B).

Although the cost per patient tested increased linearly along the prevalence of CAD (Figure 2A), both cost-effectiveness (cost per effect) and utility (cost per utility unit) showed a hyperbolic decrease in costs (Figure 2B and 2C). The observation that the effectiveness as well as the utility criterion yielded concordant results supports the validity of our findings. The decrease in costs is due to the fact that the underlying mathematical model defines a patient accurately as having CAD as the effect and an increase in QALYs (ΔQALY) as utility [19-21]. Both effect and utility become more frequent with an increase in CAD prevalence [19-21]. Specifically, the effect (i.e., the diagnosis of CAD) was based on functional testing (CMR and SPECT) [17,25]. In contrast, invasive coronary angiography relies on morphological criteria and direct visualization of the coronary arteries and may therefore be an imperfect standard of reference. Obviously, invasive fractional flow reserve is the most accurate parameter to assess the functional relevance of a stenosis, but those data were not available from CE-MARC or other large comparative studies of CMR and SPECT [17,47]. However, as coronary stenoses not causing ischemia may be judged as significant on the basis of angiographic severity alone, the actual diagnostic accuracy and therefore the cost-effectiveness of CMR is probably even better than simulated by our model.

At low CAD prevalences, both non-invasive tests were more cost-effective than invasive coronary angiography (Figure 2A and 2B). This is because the majority of negative CMR and SPECT examinations will correctly rule out significant CAD at low disease prevalences and will therefore reduce the number of invasive angiographies [18]. At higher disease prevalences, though, both non-invasive tests (SPECT > CMR) start to miss patients who actually have CAD. The consequences of such false-negative test results decrease cost-effectiveness and utility because they may prevent patients having CAD from receiving adequate treatment. The lack of treatment may lead to increased mortality, decreased quality of life, and to additional costs related to complications of CAD (i.e., treatment of myocardial infarction). False-positive test results also lead to decreased cost-effectiveness and utility, mainly due to overtreatment and unnecessary invasive coronary angiographies.

In parallel, invasive coronary angiography as the initial test becomes more competitive in terms of cost per effect and cost per utility. Sensitivity analyses indicated that above a threshold value of CAD prevalence of 0.60, performing invasive angiography was the most cost-effective strategy. This threshold is in line with previous cost-effectiveness analyses examining non-invasive strategies to detect CAD [19-21,48,49]. In addition, recent guidelines recommend invasive angiography as the most cost-effective first test if the pretest probability of CAD is >61% [50].

Our study has some limitations. Firstly, our model necessarily simplifies some aspects of the underlying clinical reality and does not account for all complications associated with CMR (e.g. gadolinium-associated nephrogenic systemic fibrosis) or SPECT (e.g. radiation-induced malignancies) [23,49]. Secondly, diagnostic accuracy data were derived from studies with an intermediate prevalence of CAD. Extrapolation of these data to populations with high or low disease prevalences should be judged with caution. Thirdly, the analysis of further imaging modalities (e.g. computed tomography or stress echocardiography) was beyond the scope of the present study. Furthermore, coronary revascularization was not within the scope of the current analysis.

## Conclusions

In patients with low to intermediate pretest probabilities, CMR is more cost-effective for the detection of CAD than SPECT. The superior diagnostic accuracy of CMR also leads to an improved clinical utility as indicated by lower costs per number of QALYs gained. Above a threshold value of CAD prevalence of 0.60, proceeding directly to invasive angiography was found to be the most cost-effective diagnostic strategy. Generally, an intermediate pretest likelihood ranges from 20-80% and there may be situations in which the likelihood of CAD exceeds 0.60, and CMR is no longer cost-effective. Thus, the most crucial step for physicians in selecting the appropriate diagnostic strategy in patients with suspected CAD is based on disease likelihood as estimated by clinical symptoms and the presence of cardiovascular risk factors.

## Competing interests

A. W. Leber and M. Dorenkamp have received lecture and consulting fees from Siemens Healthcare and were employees of Siemens Healthcare until 2009. A. Huppertz is Associate Director of the Imaging Science Institute Charité Berlin. The institute is a scientific cooperation between the Charité, University Hospital Berlin, Germany and Siemens Healthcare in form of a public-private partnership and A. Huppertz is a full-time employee of Siemens AG since June 1, 2004.

## Authors’ contributions

JB was responsible for data collection, conducted literature searches, performed data analysis and drafted and critically revised the manuscript. KB, CS, MS, and AH conducted literature searches, participated in data collection, provided technical support and revised the intellectual content of the draft. AWL and WH were responsible for conception and design, critical revision of the manuscript and provision of supervision. MD contributed to conception and design, contributed to data collection, performed statistical analyses, and critically revised the manuscript. All authors read and approved the final manuscript.
